# Gene expression signature predicts rate of type 1 diabetes progression

**DOI:** 10.1016/j.ebiom.2023.104625

**Published:** 2023-05-22

**Authors:** Tomi Suomi, Inna Starskaia, Ubaid Ullah Kalim, Omid Rasool, Maria K. Jaakkola, Toni Grönroos, Tommi Välikangas, Caroline Brorsson, Gianluca Mazzoni, Sylvaine Bruggraber, Lut Overbergh, David Dunger, Mark Peakman, Piotr Chmura, Søren Brunak, Anke M. Schulte, Chantal Mathieu, Mikael Knip, Riitta Lahesmaa, Laura L. Elo, INNODIA Consortium, Chantal Mathieu, Pieter Gillard, Kristina Casteels, Lutgart Overbergh, David Dunger, Chris Wallace, Mark Evans, Ajay Thankamony, Emile Hendriks, Sylvaine Bruggraber, Loredana Marcoveccchio, Mark Peakman, Timothy Tree, Noel G. Morgan, Sarah Richardson, John A. Todd, Linda Wicker, Adrian Mander, Colin Dayan, Mohammad Alhadj Ali, Thomas Pieber, Decio L. Eizirik, Myriam Cnop, Søren Brunak, Flemming Pociot, Jesper Johannesen, Peter Rossing, Cristina Legido Quigley, Roberto Mallone, Raphael Scharfmann, Christian Boitard, Mikael Knip, Timo Otonkoski, Riitta Veijola, Riitta Lahesmaa, Matej Oresic, Jorma Toppari, Thomas Danne, Anette G. Ziegler, Peter Achenbach, Teresa Rodriguez-Calvo, Michele Solimena, Ezio E. Bonifacio, Stephan Speier, Reinhard Holl, Francesco Dotta, Francesco Chiarelli, Piero Marchetti, Emanuele Bosi, Stefano Cianfarani, Paolo Ciampalini, Carine De Beaufort, Knut Dahl-Jørgensen, Torild Skrivarhaug, Geir Joner, Lars Krogvold, Przemka Jarosz-Chobot, Tadej Battelino, Bernard Thorens, Martin Gotthardt, Bart O. Roep, Tanja Nikolic, Arnaud Zaldumbide, Ake Lernmark, Marcus Lundgren, Guillaume Costacalde, Thorsten Strube, Anke M. Schulte, Almut Nitsche, Mark Peakman, Jose Vela, Matthias Von Herrath, Johnna Wesley, Antonella Napolitano-Rosen, Melissa Thomas, Nanette Schloot, Allison Goldfine, Frank Waldron-Lynch, Jill Kompa, Aruna Vedala, Nicole Hartmann, Gwenaelle Nicolas, Jean van Rampelbergh, Nicolas Bovy, Sanjoy Dutta, Jeannette Soderberg, Simi Ahmed, Frank Martin, Esther Latres, Gina Agiostratidou, Anne Koralova, Ruben Willemsen, Anne Smith, Binu Anand, Vipan Datta, Vijith Puthi, Sagen Zac-Varghese, Renuka Dias, Premkumar Sundaram, Bijay Vaidya, Catherine Patterson, Katharine Owen, Colin Dayan, Barbara Piel, Simon Heller, Tabitha Randell, Tasso Gazis, Elise Bismuth Reismen, Jean-Claude Carel, Jean-Pierre Riveline, Jean-Francoise Gautier, Fabrizion Andreelli, Florence Travert, Emmanuel Cosson, Alfred Penfornis, Catherine Petit, Bruno Feve, Nadine Lucidarme, Emmanuel Cosson, Jean-Paul Beressi, Catherina Ajzenman, Alina Radu, Stephanie Greteau-Hamoumou, Cecile Bibal, Thomas Meissner, Bettina Heidtmann, Sonia Toni, Birgit Rami-Merhar, Bart Eeckhout, Bernard Peene, N. Vantongerloo, Toon Maes, Leen Gommers

**Affiliations:** a Turku Bioscience Centre, University of Turku and Åbo Akademi University, FI-20520, Turku, Finland; b InFLAMES Research Flagship Center, University of Turku, Turku, Finland; c Turku Doctoral Programme of Molecular Medicine, University of Turku, Turku, Finland; d Novo Nordisk Foundation Center for Protein Research, Faculty of Health and Medical Sciences, University of Copenhagen, Copenhagen, Denmark; e Department of Paediatrics, University of Cambridge, Cambridge, England, UK; f Katholieke Universiteit Leuven/Universitaire Ziekenhuizen, Leuven, Belgium; g Immunology & Inflammation Research Therapeutic Area, Sanofi, MA, USA; h Sanofi-Aventis Deutschland GmbH, Frankfurt, Germany; i Paediatric Research Centre, University of Helsinki and Helsinki University Hospital, Helsinki, Finland; j Research Program for Clinical and Molecular Metabolism, Faculty of Medicine, University of Helsinki, Helsinki, Finland; k Tampere Centre for Child Health Research, Tampere University Hospital, Tampere, Finland; l Institute of Biomedicine, University of Turku, FI-20520, Turku, Finland

**Keywords:** Type 1 diabetes, Autoantibodies, RNA-seq, Gene expression signature, Predictive model

## Abstract

**Background:**

Type 1 diabetes is a complex heterogenous autoimmune disease without therapeutic interventions available to prevent or reverse the disease. This study aimed to identify transcriptional changes associated with the disease progression in patients with recent-onset type 1 diabetes.

**Methods:**

Whole-blood samples were collected as part of the INNODIA study at baseline and 12 months after diagnosis of type 1 diabetes. We used linear mixed-effects modelling on RNA-seq data to identify genes associated with age, sex, or disease progression. Cell-type proportions were estimated from the RNA-seq data using computational deconvolution. Associations to clinical variables were estimated using Pearson's or point-biserial correlation for continuous and dichotomous variables, respectively, using only complete pairs of observations.

**Findings:**

We found that genes and pathways related to innate immunity were downregulated during the first year after diagnosis. Significant associations of the gene expression changes were found with ZnT8A autoantibody positivity. Rate of change in the expression of 16 genes between baseline and 12 months was found to predict the decline in C-peptide at 24 months. Interestingly and consistent with earlier reports, increased B cell levels and decreased neutrophil levels were associated with the rapid progression.

**Interpretation:**

There is considerable individual variation in the rate of progression from appearance of type 1 diabetes-specific autoantibodies to clinical disease. Patient stratification and prediction of disease progression can help in developing more personalised therapeutic strategies for different disease endotypes.

**Funding:**

A full list of funding bodies can be found under Acknowledgments.

**Research in context**

**Evidence before this study**

The rate of post-onset beta cell decline during type 1 diabetes progression varies between individuals. Large scale longitudinal transcriptomics studies analysing the progression of type 1 diabetes post-onset are required to dissect the disease heterogeneity. So far, only a single study by Dufort et al. analysed longitudinal samples by RNA-seq from 138 subjects and found higher B cell levels and lower neutrophil levels to be associated with rapid loss of insulin secretion.

**Added value of this study**

By analysing whole blood RNA-seq data from 92 subjects, we confirmed increased B cell levels and decreased neutrophil levels in rapid progressors. Further, this study identified a gene expression signature that can predict type 1 diabetes progression, and suggests associations between gene expression changes and ZnT8A autoantibody positivity. Overall, understanding type 1 diabetes progression improves our ability to prevent, diagnose, and treat the disease, leading to better outcomes for patients.

**Implications of all the available evidence**

Increasing evidence suggests that type 1 diabetes is a heterogeneous disease with several disease endotypes. Identifying the signatures associated with endotypes such as rapid and slow progressors will help in patient stratification and identification of a more homogenous population for clinical trials and therapies. One benefit of a predictive signature would be the ability to intervene earlier in the disease process. This could help slow the progression of the disease and potentially prevent or delay the onset of symptoms. Another benefit would be improved monitoring of the disease progression, which would allow for more personalised treatment plans and better outcomes for patients. Finally, the gene expression signature could be used to identify new therapeutic targets to treat type 1 diabetes. Understanding the underlying mechanisms of the disease would allow developing new treatments that target specific pathways or genes involved in the disease progression.

## Introduction

Type 1 diabetes is a multifactorial autoimmune disease with genetic and environmental components. Although progress has been made, no therapies are available to prevent or reverse the disease. The development of effective therapies is hampered by our poor understanding of the pathogenesis and heterogeneity of the disease and lack of disease biomarkers and stratifiers.

Type 1 diabetes is a heterogeneous disease. There is variation in the rate of progression from appearance of type 1 diabetes-specific autoantibodies to clinical disease and in post-onset beta cell decline. The age at the diagnosis is associated with the rate of decline in insulin secretion (i.e., younger age groups decline faster).^1^^,^^2^ At least in some patients, a significant proportion of beta cells exist at diagnosis, suggesting a tempting possibility of interventions in such patients.^3^^,^^4^ Clearly, the ability to stratify patients and predict disease progression would help in developing more personalised therapies.

A specific gene expression signature might predict disease progression and monitor therapies. Gene expression changes in whole blood of children progressing to the disease occur before autoantibodies appear.^5^, ^6^, ^7^ Intriguingly, an immune cell type gene signature in patients with newly diagnosed type 1 diabetes correlates with the disease outcome.^8^

**Fig. 1:**
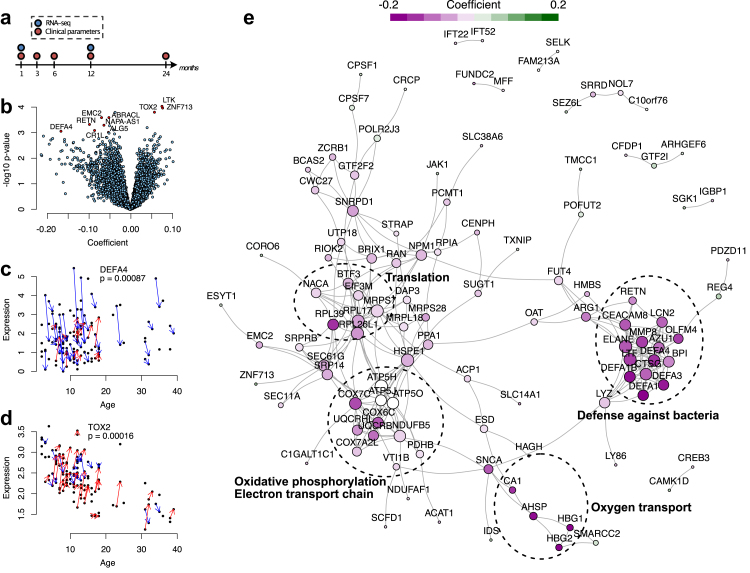
Linear mixed effects modelling of the type 1 diabetes follow-up data. (**a**) A schematic diagram of the study. Whole-blood PAXgene samples were available from the baseline and 1-year follow-up visits. (**b**) Volcano plot of the model coefficients (x-axis) and the corresponding p-values (y-axis) (n = 94; 46 with both visits). (**c**, **d**) Expression levels of *DEFA4* and *TOX2* in the cohort over time (n = 94; 46 with both visits). The baseline and 1-year follow-up samples of the same individual are connected by blue (downregulation) or red (upregulation) arrows. (**e**) STRING network with the colours representing the mixed effects model coefficients.

The aim of this study was to identify changes in whole-blood gene expression associated with disease progression in patients with recent-onset type 1 diabetes during years 1 and 2 of follow-up after the diagnosis. We used RNA-sequencing (RNA-seq)-based transcriptome analysis of peripheral blood at diagnosis and at 1 year follow-up, and correlated gene expression changes with clinical measures during disease progression (Fig. 1a). We identified a 16-gene signature that could predict disease progression. Identifying such a predictive gene signature would help to stratify patients for more personalised clinical and therapeutic interventions.

## Methods

### Clinical cohort

Whole-blood samples were collected as part of the INNODIA.^9^ The samples analysed were from the first 100 newly diagnosed INNODIA patients, consisting of 52 males and 48 females. Further details of the cohort are provided in Table S1. The cohort for this study was selected in February 2019, based on the longitudinal clinical information (up to the 6 months visit at the time), positivity for at least one type 1 diabetes–related autoantibody, gender distribution, and sample availability. The autoantibody status (IAA, GADA, IA-2A, ZnT8A) of the cohort patients indicated that three cases were negative for all of them. These cases were excluded from analysis described here. Further, genetic data defined one MODY10 case who was also removed. The final cohort consisted of 94 patients with an average age at diagnosis of 13.2 years (SD 8.5), and a disease duration of 3.9 weeks (SD 1.5) at baseline. Samples were collected at baseline, i.e., within 6 weeks of diagnosis (n = 92) and 12 months after diagnosis (n = 49) with 46 patients having samples at both time points. C-peptide, glucose, HbA1c and islet-related autoantibody measurements were carried out.^9^ Glucose was analysed locally by each site using the available hospital glucose analysis method. However, they were all performed in certified hospital labs. Harmonised protocols for sample collection and storage were used in the study clinics. The generated data is person-sensitive and access can be provided by application to the INNODIA Data Access Committee.

### Ethics

The study followed the guidelines of the Declaration of Helsinki for research on human participants, and the study protocol was approved by the ethical committees of the participating hospitals. Participants gave written informed consent.

### RNA sequencing

Prior to RNA extraction, frozen whole-blood PAXgene samples were thawed at room temperature for 2 h and subjected to RNA extraction using PAXgene Blood miRNA Kit (PreAnalytix/QIAGEN, Cat# 763134). Total RNA, including RNA longer than approximately 18 nucleotides, was purified, following the protocol supplied by the kit manufacturer. Sample concentration was measured with Nanodrop 2000 spectrophotometer and Qubit Fluorometric Quantitation (Thermo Fisher Scientific). The quality of the samples was ensured with Experion Automated Electrophoresis System (Bio Rad) and Agilent 2100 Bioanalyzer RNA Pico chip. Library preparation and sequencing were carried out at the Finnish Functional Genomics Centre (FFGC). Before starting library preparation, ERCC Spike-in Mix 1 (Invitrogen P/N 4456739) was added to 100 ng RNA according to the kit's protocol. RNA-seq libraries were prepared using TruSeq stranded mRNA HT kit and protocol # 15031047 (Illumina). The quality and quantity of the amplified libraries were measured using Advanced Analytical Fragment Analyzer (Agilent) and Qubit Fluorometric Quantitation, respectively. Pooled libraries were sequenced on an Illumina NovaSeq 6000 instrument, using 2 × 50 bp paired-end sequencing.

### RNA-seq pre-processing

Three haemoglobin-related genes, HBA1, HBA2, HBB, were overexpressed in the data with around 11% of all reads. These reads were filtered prior to normalisation. The filtered data were CPM (counts per million) scaled with TMM (Trimmed Mean of the M-values) normalisation factors, and log_2_ transformed using the R package edgeR.^10^ Extra samples included as internal quality controls and one individual classified as having *maturity onset diabetes of the young type 10* (MODY10) were excluded from the analysis. To filter lowly expressed genes, only those genes that had an average CPM >1, were included in the analysis. No imputation was performed in any of the analyses.

### C-peptide decline as a measure of disease progression

Fasted C-peptide/glucose ratio was used to measure disease progression, because mixed meal tolerance test (MMTT) data was not available for participants aged 5 years or younger and would have reduced the number of participants that could be included in the analyses. Baseline C-peptide corrected for baseline glucose has been suggested as a suitable surrogate of MMTT AUC.^11^ In line with this, we observed a high correlation among individuals with both measures available (Pearson correlation 0.96, p < 0.001; Fig. S1).

### Statistical analysis

A linear mixed effects model was fitted to the data separately for each gene, with the gene expression level as the dependent variable. Visit, sex, and age were treated as fixed effects and individual, sequencing pool and study site as random effects. The mixed effects modelling was implemented using lmerTest R package. Benjamini-Hochberg adjusted p-values were used to correct for multiple testing. We also tested linear mixed effects models with and without body mass index (BMI) as a covariate. The model with BMI had significantly better fit (lower Akaike information criterion AIC and a Chi-squared test p < 0.05 between the models) for only 3% of the genes that were included in the analysis (450 out of 13,558 genes). Overall, the results with or without BMI were highly similar (Fig. S2). The top 10 differentially expressed genes (p < 0.001 and coefficient for the visit > |0.05|) remained similar with and without BMI included in the analysis. To make the model generally applicable, we considered only the model without BMI for all genes.

A large number of different comorbidities and their combinations were also observed, but only few individuals shared the same comorbidities. This makes the inclusion of comorbidities in the models challenging, as increasing the number of covariates in the model increases the complexity of the model and, thereby, the risk of overfitting. Therefore, we have decided not to include the comorbidities in our model.

Proteins encoded by 187 differentially regulated genes between visits (Table S2) were used as an input to the STRING database (https://string-db.org/, 10th of September 2021). Both experimentally verified interactions and predicted interactions were included. Combined confidence threshold for interaction was 0.4 (medium confidence). Gene Ontology analysis was performed at http://geneontology.org. The analysis was performed separately for the upregulated and the downregulated genes. Pathways were considered to be significantly enriched at FDR <0.05 (Fisher's exact test).

Cell-type proportions were estimated from the RNA-seq data using the computational deconvolution method EPIC.^12^ The signature matrix was constructed using the publicly available human immune cell dataset available in Gene Expression Omnibus (GEO) with accession number GSE60424^13^ and the online tool CIBERSORTx.^14^ The dataset contains TMM-normalised RNA-seq data from FACS-sorted whole-blood samples with neutrophils, monocytes, B cells, CD4^+^ T cells, CD8^+^ T cells, and NK cells across different diseases.

Uniform manifold approximation and projection^15^ was applied to the gene ratios between baseline and 1-year follow-up visit, implemented using the uwot R package. The number of neighbours was set to 15, and the minimum distance was set to 0.001. Associations of the gene expression ratios to clinical variables were estimated using Pearson's or point-biserial correlation for continuous and dichotomous variables, respectively, using only complete pairs of observations. For clinical data, glycated haemoglobin (HbA1c) measurements higher than 100 were discarded as outliers. Ranked gene set enrichment analyses were performed for the results against the Hallmark sets from Molecular Signature Database (MSigDB, version 6.2) using R package fgsea, with p-value <0.01 considered significant.

Individuals were classified as rapid and slow progressors based on the change in their fasted C-peptide/glucose ratio between baseline and 2-year follow-up visits. The individuals were divided into three clusters with hierarchical clustering using Euclidean distances and the complete linkage approach. The group with the largest decrease in their ratios (<−30) was considered as rapid progressors, and the group with increase in their ratios (>5) as slow progressors. The rest of the individuals were classified as intermediate. Gene expression ratios between baseline and 1-year follow-up visits were calculated and their differences between the *rapid* and *slow* groups were tested using reproducibility optimized test statistic.^16^ Paired test was used, number of bootstrap and permutation samplings was set 1000, and the number of top list size to be considered was set as 10,000. The score for each individual was defined using the 16 differentially expressed genes (p < 0.01) and calculated as the difference between mean expression of downregulated genes and mean expression of upregulated genes, following a similar procedure as in.^17^

### Validation data

Validation data were downloaded from Gene Expression Omnibus with accession number GSE124400. Samples that did not pass the QC threshold were removed.^8^ The data were CPM-scaled with TMM-normalisation factors and log_2_-transformed using the edgeR R package.^10^ Genes with mean CPM <1 across the samples were filtered out. For validation, gene expression ratios between the baseline and 1-year measurement (±30 days) were calculated. A total of 57 patients had transcriptomics data from comparable timepoints. Similarly, as with the discovery data, mean ratios of downregulated signature genes minus mean ratios of upregulated genes were defined as a score per individual, using the 16 signature genes from the discovery cohort.

Changes in AUC C-peptide levels from 2-h mixed-meal tolerance test between the baseline and 2-year follow-up visits were estimated using the first and last available AUC C-peptide measurement. The individuals were divided into six groups with hierarchical clustering using Euclidean distances and Ward's linkage approach. Two clusters with the largest decrease over time were considered as rapid progressors, and two clusters with the smallest decrease as slow progressors.

### Role of funders

The funders had no role in the study design, data collection, analysis and interpretation of data, in the writing of the article, or in the decision to submit the paper for publication.

## Results

### Evolution of whole-blood transcriptome during type 1 diabetes progression

To identify gene expression changes during the first year after diagnosis, we performed RNA-seq of 141 whole-blood samples collected at diagnosis (baseline samples, n = 92) and at a 1-year follow-up visit (n = 49) (Fig. 1a). Linear mixed effects modelling of the RNA-seq data revealed 187 differentially expressed genes between the baseline and 1-year follow-up (Fig. 1b, Table S2). Of the top 10 differentially expressed genes (p < 0.001 and coefficient > |0.05|), seven (*DEF4A*, *RETN*, *CR1L*, *EMC2*, *ALG5*, *ABRACL, NAPA-AS1*) were downregulated, and three (*ZNF713*, *LTK*, *TOX2*) were upregulated in the follow-up samples (Fig. 1c and d: illustrating some examples).

To identify biological functions among the differentially expressed genes, we performed separate Gene Ontology (GO) enrichment analysis for the upregulated (n = 61) and downregulated (n = 126) genes. None of the terms was enriched among the upregulated genes. However, analysis of the downregulated genes revealed 16 enriched terms, including pathways involved in the immune response to bacteria, peptide biosynthetic process, and oxidative phosphorylation (OXPHOS) (Table 1).

**Table 1:** Functional enrichment analysis of the downregulated genes (n = 126) in patients with recent-onset type 1 diabetes during the first year of follow-up ranked by fold enrichment.

GO biological process term		Fold enrichment	FDR
GO:0051673	membrane disruption in other organism	66.98	0.000848
GO:0070944	neutrophil-mediated killing of bacterium	55.81	0.016200
GO:0002227	innate immune response in mucosa	36.4	0.000628
GO:0019731	antibacterial humoral response	23.92	0.000016
GO:0050832	defense response to fungus	19.7	0.000863
GO:0050829	defense response to Gram-negative bacterium	16.38	0.000028
GO:0006119	oxidative phosphorylation	16.3	0.000003
GO:0042775	mitochondrial ATP synthesis coupled electron transport	15.76	0.000143
GO:0019646	aerobic electron transport chain	14.65	0.000799
GO:0009205	purine ribonucleoside triphosphate metabolic process	13.72	0.015900
GO:0061844	antimicrobial humoral immune response mediated by antimicrobial peptide	11.49	0.002140
GO:0050830	defense response to Gram-positive bacterium	11.38	0.002200
GO:0071222	cellular response to lipopolysaccharide	6.44	0.040000
GO:0022618	ribonucleoprotein complex assembly	6.3	0.043800
GO:0043043	peptide biosynthetic process	4.32	0.039600
GO:0010467	gene expression	2.19	0.044500

To determine whether proteins encoded by the differentially expressed genes are functionally related to each other and if they form modules of functionally related genes, we formed a network of these genes taking the protein–protein functional interaction data from STRING database.^18^ Of the 187 differentially expressed genes, 110 were found to have one or more interactions in the STRING database (Fig. 1e). The molecular complex detection algorithm MCODE^19^ identified four modules in the network, which were related to defense against bacteria, translation, OXPHOS, and oxygen transport. All the genes in the “defense against bacteria” and “oxygen transport” modules had reduced expression over time, suggesting that they were downregulated as the disease progressed (Fig. 1e, Table S2).

### Association of gene expression changes with the types of autoantibodies

**Fig. 2:**
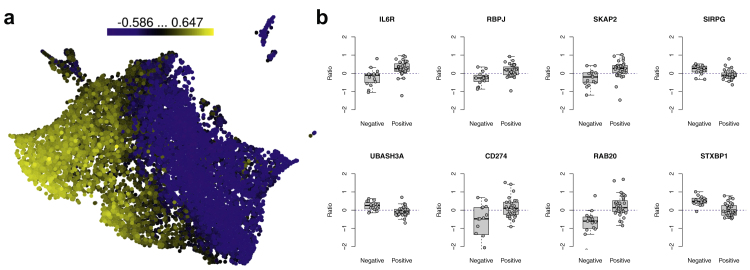
Correlations of gene expression ratios between the baseline and 1-year follow-up samples (i.e., expression change) against zinc transporter 8 (ZnT8) autoantibody status at baseline. (**a**) Uniform manifold approximation and projection dimensional reduction of all gene ratios (n = 46), coloured on the basis of the correlation. (**b**) Examples of gene ratios between the baseline and 1-year follow-up samples for ZnT8-autoantibody positive (n = 31) and negative (n = 15) individuals.

We next assessed whether gene expression correlated with types of autoantibodies (IAA, GADA, IA-2A, and ZnT8A) detected at the baseline visit. We found significant associations between changes in gene expression and ZnT8A autoantibody positivity (FDR < 0.05, Table S3) but not with other autoantibodies. Among the patients, 64 tested positive for ZnT8A antibodies, and 421 genes showed point-biserial correlation (FDR < 0.05, |r| > 0.4) with ZnT8A autoantibody positivity (Fig. 2a, Table S3). Among these genes, five (*IL6R*, *RBPJ*, *SKAP2*, *SIRPG*, *UBASH3A*) harbour a nearby type 1 diabetes-associated SNP.^20^ Further, *IL6R*, *RBPJ*, *SKAP2, CD274* and *RAB20* correlated positively with the ZnT8A autoantibody positivity (i.e., their expression increased in individuals positive for ZnT8A) while it decreased in ZnT8A-negative patients at the follow-up timepoint (Fig. 2b). On the other hand, *SIRPG*, *UBASH3A* and *STXBP1* had inverse correlations (Fig. 2b).

### Association of gene expression changes with C-peptide levels

**Fig. 3:**
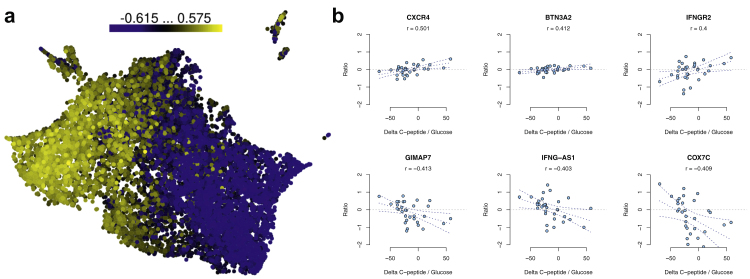
Correlations of gene expression ratios between the baseline and 1-year follow-up samples (i.e., expression change) against changes in C-peptide/glucose ratio between baseline and follow-up. (**a**) Uniform manifold approximation and projection (UMAP) of all gene ratios (n = 46). (**b**) Scatterplots of selected gene ratios with C-peptide/glucose ratios (n = 32).

We next determined whether gene expression changes during the 1-year follow-up correlated with a clinical measure of disease progression (i.e., C-peptide/glucose ratio) (Fig. 3a). We used the change in fasted C-peptide between the baseline and 2-year follow-up visit, instead of 1-year follow-up visit, as a measure of rate of decline in C-peptide because in some patients, partial remission (also known as “honeymoon phase”) lasts 3–9 months after starting insulin therapy,^21^ making 1-year C-peptide measurements less stable. Indeed, for some individuals, the fasted C-peptide levels varied considerably between the 1- and 2-year follow-up visits: 392 genes were associated (p < 0.05, |r| > 0.4) with the change in fasted C-peptide/glucose ratio between the visits (Fig. 3a, Table S4).

Three genes positively associated with the change in fasted C-peptide/glucose ratio are particularly noteworthy (Fig. 3b, upper panel). CXCR4 and its ligand CXCL12 are important in autoimmune diabetes in mice.^22^ In addition, CXCR4 in B cells and CD4^+^ T cells was upregulated more in patients with systemic lupus erythematosus than in controls.^23^ *BTN3A2,* a gene of the extended class I region of the major histocompatibility, is associated with type 1 diabetes.^24^ It was upregulated in peripheral blood mononuclear cells of children at risk of the disease before the appearance of type 1 diabetes-associated autoantibodies.^25^ *IFNGR2* encodes the non-ligand-binding beta chain of the human interferon gamma receptor heterodimer.

Genes negatively associated with the change in fasted C-peptide/glucose ratio were also of interest (Fig. 3b, lower panel). *GIMAP7* was upregulated in peripheral blood CD8^+^ T cells before any autoantibodies appeared in children at risk.^25^ A long, non-coding RNA *IFNG-AS1* enhances IFNG expression in NK cells.^26^ The association of C-peptide decline with *IFNG-AS1* and *IFNGR2* suggests a link with IFNγ signalling and disease progression.

### Gene expression signature predicting C-peptide decline

**Fig. 4:**
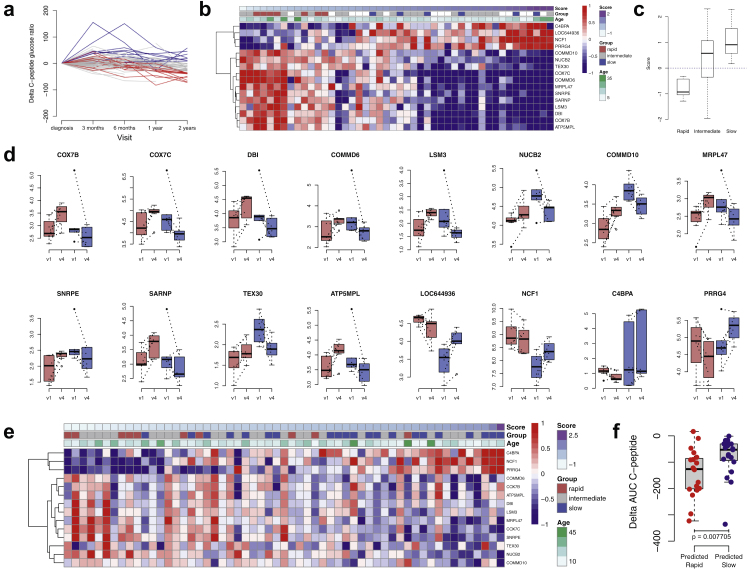
Predictive model for C-peptide decline. (**a**) Delta fasted C-peptide/glucose ratio at each sampling timepoint (as outlined in Fig. 1). Grouping of individuals was based on changes up to the 2-year follow visit. Blue and red lines show the rapid and slow progressors, respectively, and grey lines show the patients of intermediate category. (**b**) Heatmap of genes with differential gene expression ratios between rapid (n = 7) and slow (n = 6) groups by ROTS across the individuals (n = 46). (**c**) Signature score for individuals in rapid (n = 7), intermediate (n = 19), and slow (n = 6) groups. (**d**) Individual gene expression changes in the signature genes between baseline (v1) and 1-year follow-up visits (v4) for rapid (n = 7) and slow (n = 6) progressors. (**e**) Heatmap of signature gene ratios in validation data (n = 57) annotated with delta AUC C-peptide based rapid (n = 10) and slow (n = 21) progressors. (**f**) AUC C-peptide changes in the validation data for predicted rapid (n = 19) and slow (n = 19) groups.

Next, we examined whether alterations in gene expression during the first follow-up year predict the rate of disease progression at 2 years after the diagnosis. We stratified the patients into three groups (Fig. 4a): those with an increase of >5 in their fasted C-peptide/glucose ratio between the baseline and the 2-year visits were considered as slow progressors (n = 8), and those with a decrease of >30 were considered rapid progressors (n = 13). The rest of the patients represented the intermediate category. Out of these, 7 patients in the rapid group and 6 patients in the slow group had gene expression data both from the baseline and 1-year follow-up visits. Statistical testing using reproducibility-optimized test statistic^16^ between the rapid and slow groups revealed 16 signature genes (Fig. 4b). A prognostic score calculated as in our previous study,^17^ using the transcriptomics data at baseline and 1-year, predicted the rate of disease progression at two years (Fig. 4c). Interestingly, individual gene expression changes between baseline and 1-year follow-up visit for the rapid and slow progressors (Fig. 4d), show that changes in expression (either up or down) of the signature genes display opposite expression profiles in the two patient groups, and not just have different rates of change to the same direction.

To validate our model in an independent cohort of patients affected by type 1 diabetes, we used published data on the whole-blood transcriptome analysis of 137 new-onset type 1 diabetes patients,^8^ of which 57 had gene expression measurements from comparable timepoints (baseline and 1-year follow-up). Based on expression of the 16 genes in our present study, we calculated the prognostic scores for the 57 patients of the validation cohort (Fig. 4e). With the 2-year delta AUC C-peptide based classification of rapid and slow progressors and using 1-year RNA-seq based score ±0.25 as a cutoff for predicting individuals to these classes, there were 7 rapid and 3 slow individuals in the predicted rapid group, and 10 slow and 1 rapid individuals in the predicted slow group. The difference in the AUC C-peptide decline between the predicted groups was statistically significant (Wilcoxon rank sum test p < 0.01, Fig. 4f).

### Neutrophils were the cell type most strongly associated with the disease progression

**Fig. 5:**
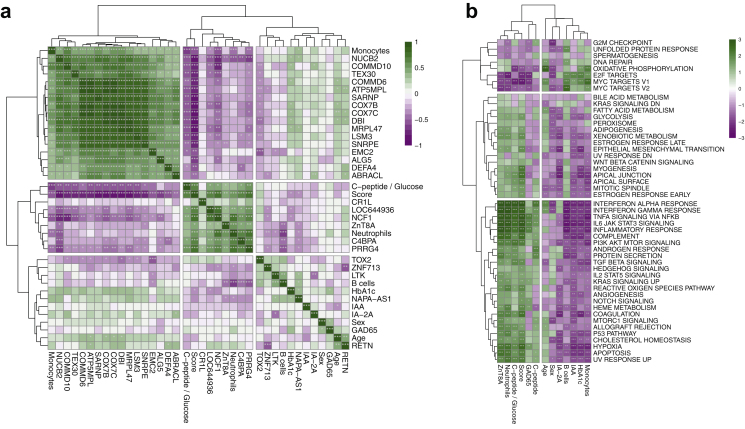
Investigation of the differentially expressed and predictive genes. (**a**) Correlations among clinical variables, top differentially expressed genes between baseline and 1-year follow-up, and predictive signature genes. (**b**) Gene set enrichment analysis on the ranked lists of genes based on their correlation with clinical parameters, cell-type proportions, and prognostic score, using Hallmark gene sets from the molecular signature database (MSigDB). The colour scale is based on normalised enrichment score, and the significance is denoted by asterisks ∗ p = 0.05, ∗∗p = 0.01, ∗∗∗p = 0.001.

Finally, we estimated the contribution of different cell types to the RNA-seq data to understand whether the cell-type proportions were different between the individuals. Reliability of the cell-type proportion estimates was assessed by comparing them with measured proportions, which were available for some samples. Measurements were reliable for neutrophils, monocytes, and B cells (Pearson correlation 0.69, 0.53, and 0.72 respectively; Fig. S3). Neutrophil abundance showed a positive correlation with the score of disease progression for 4 of the 16 signature genes: *LOC644936*, *NCF1*, *C4BPA* and *PRRG4* (Spearman correlation 0.50, 0.71, 0.65, and 0.76 respectively, p < 0.001). Monocytes showed an inverse correlation with the change in fasted C-peptide/glucose ratio and the score of disease progression (Spearman correlation −0.62 and −0.59 respectively, p < 0.001). Further, the estimated proportion of B cells revealed a strong inverse correlation with neutrophils (Spearman correlation −0.69, p < 0.001) (Fig. 5a, Fig. S4), consistent with earlier observations where high B cell proportions were associated with fast progression.

We performed gene set enrichment analysis on the ranked list of genes based on their correlation with clinical parameters, cell-type proportions, and the prognostic score, using Hallmark gene sets from molecular signature database (MSigDB).^27^ Several immune-related signalling pathways (e.g., TNFα, IL-6/JAK/STAT3, and IL-2/STAT5 signalling) were enriched among genes positively correlated with the fasted C-peptide/glucose ratio and neutrophil abundance (Fig. 5b). These pathways were not enriched among genes correlating with age or sex. Other enriched pathways included interferon alpha and gamma responses, androgen response, and protein secretion pathways, which were enriched among the genes positively correlating with fasted C-peptide/glucose. Pathways of OXPHOS and Myc targets were enriched among genes inversely correlating with the fasted C-peptide/glucose ratio. As expected, pathway enrichment showed an opposite trend with neutrophil and monocyte levels.

## Discussion

To study the disease progression after the diagnosis of type 1 diabetes, we examined gene expression changes in peripheral blood of type 1 diabetes patients during the first year after the diagnosis and studied their correlations with the fasted C-peptide/glucose ratio after 2 years. We found numerous changes 1 year after disease onset, particularly in genes related to the immune response to bacteria, oxidative phosphorylation (OXPHOS), and RNA processing and translation. Gene expression changes were associated with ZnT8A autoantibody positivity but not with the other autoantibodies. Importantly, we identified a 16-gene signature with changes between baseline and 1-year follow-up that was associated to the rate of decline in insulin secretion 2 years after diagnosis. We also assessed that gene signature in an independent published dataset. However, additional validation in a larger cohort is needed to confirm our findings.

Interestingly, the immune response to bacteria, including the neutrophil-mediated response, was among the most enriched processes among the genes downregulated when progressing to disease. Diabetes has been associated with an increased risk of infectious diseases,^28^, ^29^, ^30^ and the prevalence of bacterial infections is higher in individuals with type 1 diabetes than in their non-diabetic controls.^31^ Recent research showed neutrophils might be implicated in pathogenesis of type 1 diabetes. For instance, decreased neutrophil numbers were reported in patients with recent-onset type 1 diabetes as well as in presymptomatic autoantibody-positive individuals.^32^^,^^33^ Further, reduction of neutrophils in presymptomatic at-risk individuals and type 1 diabetes patients was associated with poor beta cell function and increased pancreatic neutrophil infiltration.^34^ In addition, the results demonstrating the role of neutrophils in initiation of beta-cell autoimmunity are consistent with the observations reported earlier in murine studies.^35^ Studies both in man and mouse^34^^,^^35^ showed that neutrophils infiltrating pancreas release neutrophil extracellular traps, which might directly lead to the disturbance of beta cell function and initiation of the disease.

Among the 16 genes predicting disease progression, *LOC644936, NCF1*, *PRRG4*, and *C4BPA,* were primarily expressed by neutrophils and correlated positively with the change in fasted C-peptide/glucose ratio. NCF1 is a part of the NOX2 complex that transfers electrons from NADPH to oxygen generating reactive oxygen species (ROS) and plays a crucial role in host defence. C4BPA inhibits the classical and lectin pathways of complement as well as phagocytosis of apoptotic cells.^36^ However, the precise role of C4BPA and PRRG4 in neutrophils is not well known. Rapid progressors have earlier been reported to have lower levels of neutrophil-related gene expression.^8^

Genes related to OXPHOS were downregulated at 1-year post-onset in our data. Further, OXPHOS genes predicted progression of the disease and were upregulated as the disease progressed in rapid progressors (Fig. 4b) while they were downregulated in slow progressors. OXPHOS efficiently generates large amounts of ATP in mitochondria. In a cross-sectional microarray study of whole blood samples, genes of this pathway were upregulated in patients with newly diagnosed type 1 diabetes compared to non-diabetic controls.^7^ According to our results, UQCRB and COX7C were downregulated at one year post onset as compared to baseline and they were upregulated in newly diagnosed type 1 diabetes patients compared to non-diabetic controls in Reynier et al. (2010) study. Interestingly, COX7C along with COX7B were among the signature genes predicting the rate of progression; both genes were upregulated during disease progression in rapid progressors but were downregulated in slow progressors. Higher expression of OXPHOS genes in rapid progressors is perhaps consistent with higher energy expenditure in C-peptide negative type 1 diabetes patients.^37^ Further work is needed to understand the regulation of OXPHOS in progression to type 1 diabetes.

Similarly, the genes associated with RNA processing and translation were downregulated 1-year post-diagnosis. Further, splicing-related genes were part of the signature predicting the rate of progression. *LSM3*, a member of the Lsm (Like Sm) protein family, is involved in pre-mRNA splicing and mRNA degradation.^38^ *SNRPE*, encoding the small nuclear ribonucleoprotein polypeptide E, is a key component of the pre-mRNA spliceosome. Similarly, *SAPNP* (also known as *CIP29*) is a ribonucleoprotein participating in mRNA splicing. Pre-mRNA splicing may contribute to the pathogenesis of type 1 diabetes by affecting splice variant expression of susceptible genes.^39^

We found positivity for ZnT8A, but not for other islet-associated autoantibodies (i.e., IAA, GADA and IA-2A) to be associated with the progression of type 1 diabetes. Lack of associations of C-peptide status with GADA, ICA and IA-2A was also reported earlier in a previous 6-year follow-up study of young adults with type 1 diabetes.^40^ A previous study showed that in children with type 1 diabetes, positivity for ZnT8A at diagnosis correlated with low C-peptide levels 2 years later, which resulted in a higher daily insulin requirement in these patients.^41^ Thus, the appearance of ZnT8A may indicate a more severe disease phenotype in children with early disease onset. However, in young adults (15–34 years old) with type 1 diabetes, high C-peptide levels at diagnosis were correlated with sustained levels of ZnT8A during the 5 subsequent years.^42^

We found *SKAP2, CD274* and *RAB20,* among others, to correlate positively with the ZnT8A autoantibody positivity. A variant in *SKAP2* was predictive of beta cell function in newly diagnosed patients.^43^ Moreover, a gain-of-function variant in *SKAP2* resulted in enhanced activity of integrin pathways and migratory phenotype of macrophages, which likely contributed to type 1 diabetes development.^44^ *CD274* encodes PD-L1, the ligand for inhibitory receptor of B7 family expressed on T cells. Under inflammatory conditions (e.g., high IFNγ), pancreatic beta cells upregulate PD-L1 expression to limit the T-cell response.^45^ RAB20, a member of the RAS oncogene family, is highly expressed in monocytes and neutrophils (Human Protein Atlas). In mouse macrophages, *RAB20* expression is induced by IFNγ in the phagosomes, a process leading to phagosome maturation delay, which is critical for efficient antigen presentation.^46^^,^^47^

On the other hand, *SIRPG, STXBP1* and *UBASH3A* had inverse correlations with the ZnT8A autoantibody positivity. The type 1 diabetes associated SNP near *SIRPG* was shown to modulate the risk of the disease by controlling the alternative splicing of the gene.^48^ It encodes syntaxin-binding protein 1, which regulates docking and fusion of vesicles with the plasma membrane during exocytosis. *STXBP1* is important in cytotoxic activity of CD8^+^ T cells and NK cells.^49^ A genetic variant in *UBASH3A* is linked to type 1 diabetes development in children from the DAISY and BABYDIAB cohorts.^50^ The type 1 diabetes-associated variants in *UBASH3A* in human CD4^+^ T cells resulted in higher levels of gene expression and decreased NF-kB signalling and *IL2* expression.^51^

We found gene expression changes in the whole blood of patients with recent-onset type 1 diabetes and identified a gene signature that predicted the rate of type 1 disease progression and validated the predictive model in an independent cohort. Given the heterogeneity of type 1 diabetes in terms of risk groups, types of autoantibodies and potential triggers and drivers of the disease, the number of individuals in the study is an important limitation and the results need to be validated in a larger cohort or with more homogenous cohort of similar size. As the study was conducted on Caucasian population it remains to be seen if the findings wil be valid also for other populations. The predictive model, if validated further, may assist in patient stratification for developing personalised therapeutic strategies.

## Contributors

INNODIA and INNODIA HARVEST provided expertise and facilitated data collection and curation. SB, LO, DD, MP, PC, SB, AMS, CM, MK, RL, LLE designed the study. TS, IS, UUK, OR, MKJ, TG, TV, CB, GM collected data and performed analyses. TS, IS, UUK, OR, RL, LLE wrote the manuscript. TS, IS, UUK, OR verified the underlying data. All authors revised the manuscript and approved the final version.

## Data sharing statement

The generated data is person-sensitive and access can be provided by application to the INNODIA Data Access Committee.

## Declaration of interests

CM serves or has served on the advisory panel for ActoBio Therapeutics, AstraZeneca, Avotres, Boehringer Ingelheim, Eli Lilly and Company, Imcyse, Insulet, Mannkind, Medtronic, Merck Sharp and Dohme Ltd., Novartis, Novo Nordisk, Pfizer, Roche, Sandoz, Sanofi, Vertex, and Zealand Pharma. CM serves or has served on the speakers bureau for AstraZeneca, Boehringer Ingelheim, Eli Lilly and Company, Novartis, Novo Nordisk, and Sanofi. “T.G. was supported by 10.13039/501100002341Academy of Finland, Tampere University and University of Turku”.
