# Supplementary figures and images for: Gene expression signature predicts rate of type 1 diabetes progression
Source: eBioMedicine. 2023 May 22;92:104625. doi: 10.1016/j.ebiom.2023.104625 (PMC10277927; doi:10.1016/j.ebiom.2023.104625)

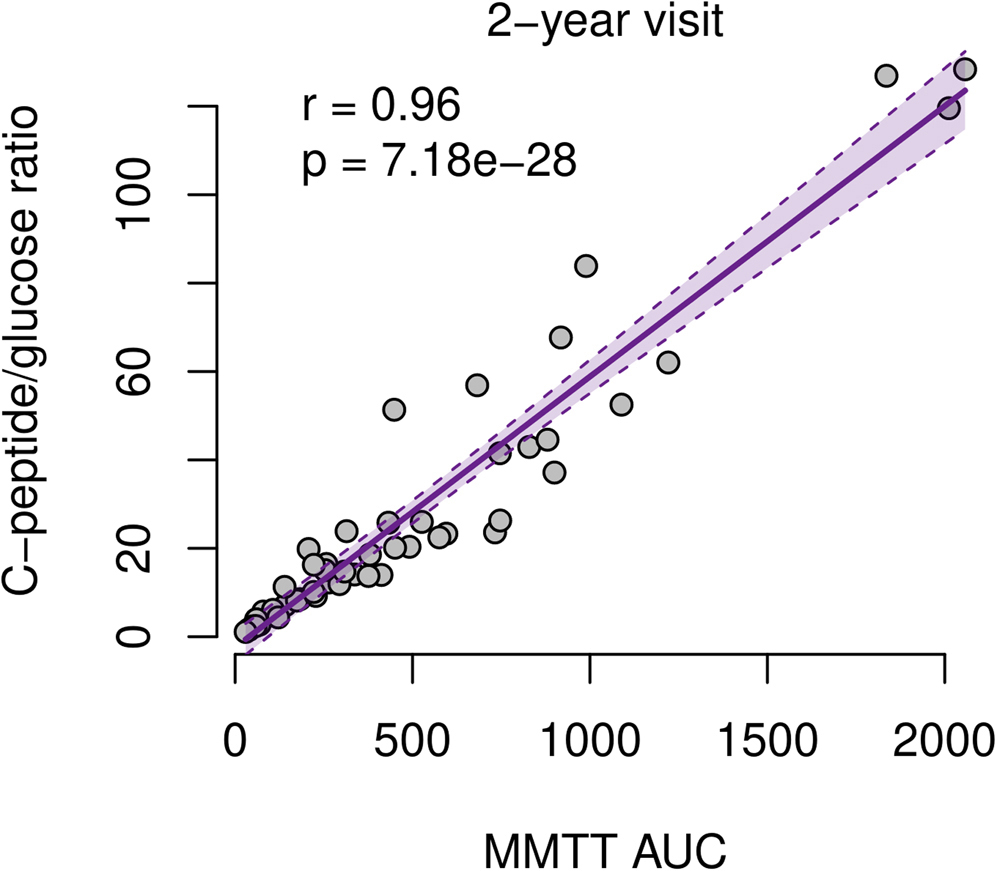

Supplement: Fig S1 [file figs1.jpg]

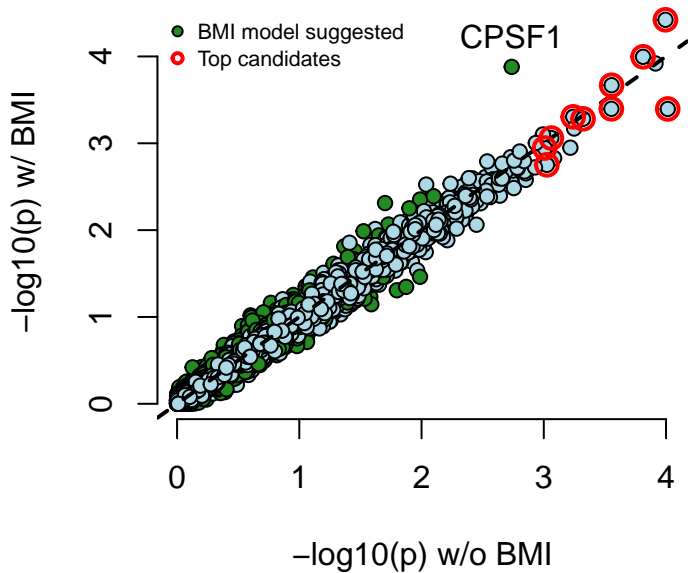

Supplement: Fig S2 [file mmc5.pdf]

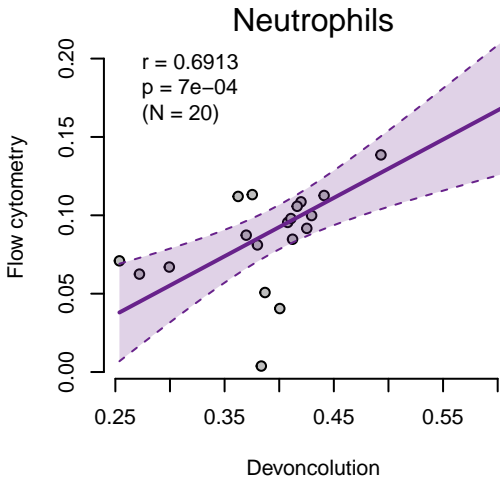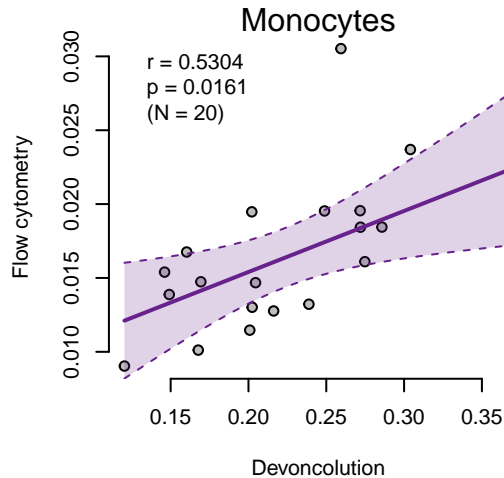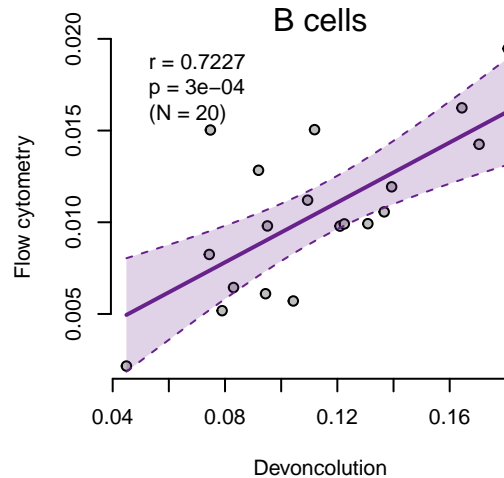

Supplement: Fig S3 [file mmc6.pdf]

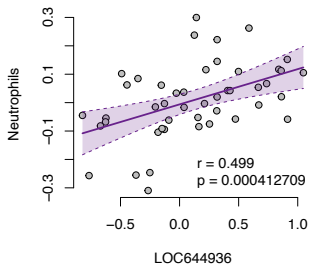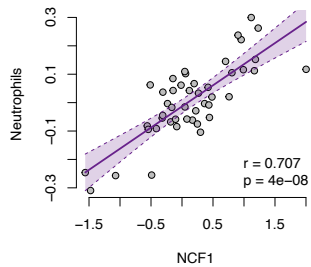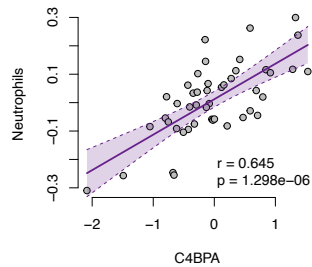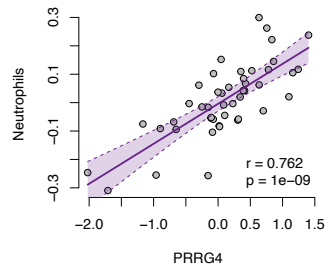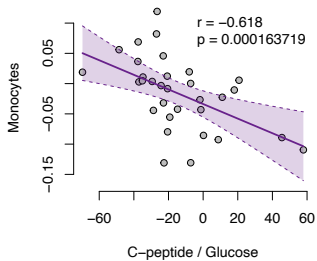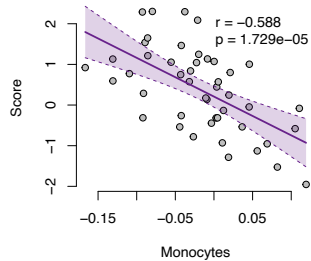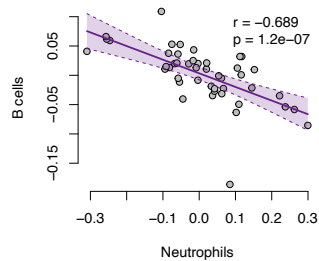

Supplement: Fig S4 [file mmc7.pdf]
